# In Vitro Exposure to Vaped Tetrahydrocannabinol Increases *Candida albicans* (SC5314) Growth, Metabolic Activity, Biofilm Formation, and the Expression of Virulence Genes

**DOI:** 10.3390/microorganisms13102278

**Published:** 2025-09-29

**Authors:** Fatima-Zahrae Laaboudi, Omayma Amri, Mahmoud Rouabhia

**Affiliations:** Groupe de Recherche en Écologie Buccale, Faculté de Médecine Dentaire, Université Laval, 2420 rue de la Terrasse, Québec QC G1V 0A6, Canada; fatima-zahrae.laaboudi.1@ulaval.ca (F.-Z.L.); omaymaa.amri@gmail.com (O.A.)

**Keywords:** e-cigarette, cannabis, vaping THC, oral infection, gene expression

## Abstract

Vaping tetrahydrocannabinol (THC), a cannabis derivative, using electronic cigarettes (e-cigarettes) could deregulate oral health and lead to oral candidiasis. This study aimed to investigate the effects of vaped THC on *Candida albicans* growth, metabolic activity, biofilm formation, and the expression of virulence genes. Exposure to e-cigarette aerosol with or without nicotine and with or without 10% or 15% THC increased *C. albicans* growth and metabolic activity; the effects were more pronounced when THC was present in the e-cigarette aerosol. Biofilm analyses showed that e-cigarette aerosol with THC significantly promoted *C. albicans* biofilm formation, with the higher THC concentration (15%) having the greater effect. Consistently, e-cigarette aerosol with THC increased the expression of the virulence genes *EAP1*, *SAP2*, *SAP4*, and *SAP9*. These findings suggested that exposure to vaped THC could contribute to the pathogenesis of oral candidiasis, which may lead to oral health problems.

## 1. Introduction

The consumption of the cannabis plant in various forms has been prohibited by legislation, yet many people still consume it. These bans are related to the physiological issues that can be caused by consuming or using cannabis and its derivatives, including mental issues that may cause a person to lose control over their actions [1,2,3]. The physiological effects evoked by cannabis are caused by its constituents, including more than a hundred phytocannabinoids [2,3]. The abundant cannabinoids in the cannabis plant are tetrahydrocannabinol (THC), cannabidiol (CBD), cannabinol (CBN), and cannabigerol (CBG) [4,5]. They are primarily present in cannabis flowers and leaves, with much lower levels in the stems and roots [4,5]. The most studied cannabinoids are CBD and THC. There are several reports that CBD does not have adverse effects when administered at specific amounts to humans to control diseases such as epilepsy [6]. CBD is also considered an efficient drug to treat anxiety, psychosis, nausea, and rheumatoid arthritis [7,8]. It also prevents/decreases THC-elicited psychotic symptoms and reduces the negative impact of THC on hippocampus-dependent memory [9]. THC is the principal psychoactive constituent of the cannabis plant [10]. It has several medical uses: its antiemetic effects are useful for patients receiving cancer chemotherapy and it serves as an appetite stimulant for people with acquired immunodeficiency syndrome (AIDS). It is also used to improve sleep [11]. Furthermore, THC has pleiotropic effects in humans, including relaxation, dysphoria, tolerance, and dependence [12].

There have been notable shifts in societal perceptions and legislation regarding the use of cannabis, leading to recreational use of cannabis products including the addition of CBD and THC oils in drinks, foods, and topical creams [13]. However, the easiest way to use cannabis is via smoking [14]. Cannabis smoke is frequently associated with cigarette smoke. Goodwin et al. (2018) estimated that 5.2% of adults use cigarettes and cannabis. In that study, 69% of cannabis users reported tobacco use, and 17.8% of cigarette users reported using cannabis [14]. Additionally, daily cannabis smokers are more likely to use combustible tobacco than non-daily cannabis smokers. Like cigarette smoke, cannabis smoke has adverse effects on the user’s health [15,16,17]. Over 17% of people who start consuming cannabis as adolescents will develop cannabis use syndrome, which includes irritability, anger, depression, difficulty sleeping, craving, and decreased appetite [18]. In the oral cavity, smoking cannabis has been associated with multiple disorders, including periodontitis, dental caries, oral candidiasis, and oral cancers [19,20,21]. Electronic cigarettes (e-cigarettes) have been developed to mitigate these adverse effects. They allow a user to vape cannabis products, including CBD and THC [22,23]. This modality has attracted the attention of adults, teenagers, and even non-formal smokers [13].

CBD and THC vaping is increasing among teenagers because it is one of the fastest ways to administer cannabis derivatives [24,25]. Advertisements of these products and their commercial availability have led to the perception that these products have a lower health risk and are more socially acceptable compared with smoking traditional cigarettes [26]. However, growing evidence indicates that there are important health concerns related to vaping cannabis derivatives [27,28,29]. Indeed, vaping THC has been associated with lung injury [27], wheezing, and dry cough [30]. At the oral level, vaping cannabis has been associated with xerostomia [28], poor periodontal health, and dental caries [29]. THC-rich e-cigarette aerosol contacts all constituents of the oral cavity, including the soft and hard tissues, as well as the buccal ecosystem. It can deregulate this ecosystem, leading to bacterial or fungal oral infections [20,31].

*C. albicans* is a commensal yeast that inhabits the oral cavity of about 70% of the population [32]. Oral hygiene conditions and the immune system control yeast pathogenesis. However, different agents, including cigarette smoke, cannabis smoke, inadequate hygiene, and oral diseases such as cancer, can promote *Candida* pathogenesis [17,33]. In recent decades, the yeast *C. albicans* has emerged as a significant public health concern, causing candidiasis. The spectrum of diseases caused by this species ranges from vaginal infections, oral and cutaneous candidiasis, to deep infections in hospitalized patients, which lead to high morbidity and mortality rates [34,35]. *C. albicans* pathogenesis involves the expression of several virulence genes, including host recognition biomolecules (adhesins) genes, morphogenesis (the transition from blastospore to hypha forms) genes, and secreted aspartyl proteases (SAP, contributing to yeast invasion of tissue) genes [36].

Oral candidiasis could also occur after vaping THC, leading to oral candidiasis. Hence, we evaluated the effects of THC-rich aerosols on the growth, biofilm formation, and expression of virulence genes in *C. albicans*.

## 2. Materials and Methods

### 2.1. Candida Strain and Culture Conditions

*C. albicans* strain SC5314 (ATCC, Manassas, VA, USA) was grown in Sabouraud Dextrose Broth (Difco Laboratories, Becton, Dickinson and Company, Mississauga, ON, Canada), supplemented with 0.1% glucose (pH 5.6). The cells were incubated for 24 h at 30 °C in a 5% CO_2_ atmosphere [37,38]. This *C. albicans* strain is genetically stable, well characterized, and frequently used to perform in vitro [39] and animal studies [40].

After incubation, the cells were counted using a hemacytometer and adjusted to 10^3^ colony-forming units (CFU)/mL before use. This strain was chosen for its significant invasive properties and virulence compared with other strains [41], its robust growth rates indicating its adaptability and resilience [40], its predominant clade association [42], and its extensively sequenced phased diploid genome [43].

### 2.2. E-Cigarettes, E-Liquids, and THC Used in the Experiments

E-cigarette devices (Joyetech eGo Pod system) were purchased from Canada Vape (www.canadavapes.com, accessed on 6 June 2024) to generate the e-cigarette aerosols. They were chosen due to their availability to e-cigarette users. These devices take a 2-mL e-liquid pod and feature draw-activated operation. Tobacco-flavoured e-liquid was purchased from an e-vapor store in Quebec City, Canada. This e-liquid contained 50% propylene glycol (PG) and 50% vegetal glycerine (VG) without nicotine or with 12 mg/mL nicotine. Cannabis oil (Sativa 750, Lot: 0001349) was purchased from the Société Québécoise du cannabis store (SQDC, Quebec City, QC, Canada). The THC content in this oil is 27 mg/g, and the CBD content is less than 1 mg/g, with no other chemicals, according to the manufacturer’s instructions. The oil was stored at 20 °C in the dark. For this study, cannabis oil (THC) was diluted in e-liquid at 10% or 15% for use in the e-cigarette devices. These THC concentrations mimic those vaped by e-cigarette users [44].

### 2.3. E-Vapor Generating System

The e-vapor generating system was established in a previous study [45], and was used with slight modifications. The e-cigarette device was placed into one end of a silicone tube linked to a peristaltic pump. The other end of the tube was connected to a custom-made vaping chamber to expose a *C. albicans* cell suspension to e-cigarette aerosol for 5, 10, or 20 min, twice a day, while maintaining mild agitation to promote uniform contact of the cells with the generated e-cigarette aerosol. The aerosol was generated via the activated peristaltic pump and injected into the vaping chamber through the silicone tube. The aerosol-generating cycle consisted of a 5-s aerosol puff followed by a 25-s pause. This exposure regimen was repeated twice per minute, as described previously [46].

### 2.4. Effect of E-Cigarette Aerosol with or Without THC on C. albicans Growth Kinetics

*C. albicans* was sub-cultured overnight at 30 °C in a 5% CO_2_ incubator. A hemacytometer was used to count the number of cells, and the cell suspension was adjusted to 10^6^ CFU/mL. This cell suspension was distributed into 50-mL tubes at 10^3^ CFU/mL (with a total volume of 5 mL per tube). The experimental groups are listed in Table 1. The cells were exposed or not exposed to e-cigarette aerosol for 5, 10, or 20 min. The exposure regimen was repeated twice, with a 4-h period between each exposure, during which the cells were incubated at 30 °C in a 5% CO_2_ atmosphere. Following the second exposure, the cell suspension was distributed at 200 µL per well in 96-well plates (24 replicates per group). Each 96-well plate was sealed with a transparent adhesive optically clear seal (Microseal ‘B’ Film, Bio-Rad, Hercules, CA, USA), and placed in a microplate reader (Promega, Madison, WI, USA) at 30 °C. The absorbance was read at 530 nm over a period of 24 h. Before each reading, the plate was shaken automatically for 5 s to ensure a homogeneous cell suspension. The absorbance was saved and used to construct cell growth kinetic curves in Microsoft Excel. The results are presented as the mean ± standard deviation (SD) of five independent experiments.

### 2.5. Effect of E-Cigarette Aerosol on Biofilm Formation

*C. albicans* can adhere to both biotic and abiotic surfaces, as well as to other cells, leading to biofilm formation. The biofilms were produced using a three-dimensional (3D) porous collagen matrix (Zimmer^®^ Collagen Tape; Zimmer Dental Inc., Carlsbad, CA, USA). Briefly, 1 cm × 1 cm samples of sterile 3D matrices were placed in wells (three replicates) of 12-well plates. Then, *C. albicans* (10^3^ CFU) in 100 µL of medium was seeded onto each 3D matrix. The plates were incubated for 60 min at 30 °C in a 5% CO_2_ atmosphere to allow *C. albicans* to adhere the 3D matrix. After this incubation, 1 mL of fresh Sabouraud medium was added to each well, and the plates were incubated for 24 h at 30 °C in a 5% CO_2_ atmosphere. After incubation, the medium was removed from each well, and the 3D matrix populated with *C. albicans* was exposed twice a day for 3 days to e-cigarette aerosol for 5, 10, or 20 min. All experimental conditions are described in Table 1.

**Table 1:** The different experimental groups to be exposed or not to e-cigarette aerosols.

Group	Exposure Condition
Control (Ctrl)	Cells not exposed to e-cigarette aerosol
Nicotine-free (vehicle)	E-cigarette aerosol generated with e-liquid containing PG and VG, only.
Nicotine-rich	Nicotine (12 mg/mL) rich e-cigarette aerosol.
Nicotine-free plus 10% THC	E-cigarette aerosol containing 10% THC.
Nicotine-rich plus 10% THC	E-cigarette aerosol containing nicotine and 10% THC
Nicotine-free plus 15% THC	E-cigarette aerosol containing 15% THC.
Nicotine-rich plus 15% THC	E-cigarette aerosol containing nicotine and 15% THC

Following each exposure to e-cigarette aerosol with or without THC, 2 mL of Sabouraud medium was added to each well, and the plates were incubated at 30 °C in a 5% CO_2_ incubator. For each day, the two exposures were separated by a 4-h incubation period. The medium was removed before each exposure to allow for maximum contact between the e-cigarette aerosol and the *C. albicans*–seeded 3D matrixes. Control groups were included for each condition, representing unexposed *C. albicans*–seeded 3D matrix. Twenty-four hours after the third exposure day, each 3D matrix was fixed overnight with 4% paraformaldehyde solution at 4 °C. Biofilm formation was evaluated using histological analysis and crystal violet staining.

#### 2.5.1. Biofilm Structure

Following paraformaldehyde fixation, the biofilms were washed with phosphate-buffered saline (PBS), dehydrated, and embedded in paraffin. Thin sections (about 12 µm) were cut from each paraffin-embedded biofilm and then stained with Masson’s trichrome. After staining, the tissue sections were observed under an optical microscope (objective 20×) and photographed. The images are representative of four independent experiments.

#### 2.5.2. Crystal Violet Staining

Briefly, biofilms were placed individually in the wells of 12-well plates, rinsed twice with PBS, and immersed in 500 µL of 0.1% crystal violet solution for 15 min under gentle shaking. Then, each 3D matrix was washed extensively with PBS to remove unfixed crystal violet dye, before drying them for 24 h under a chemical hood. To release crystal violet, each sample was layered with 2.5 mL of 30% acetic acid and incubated for 20 min under agitation. Each solution was diluted 1:3 in PBS, distributed in the wells of 96-well plates (four replicates with 200 µL per well), and the absorbance was read at 595 nm, as previously reported [47].

### 2.6. Effect of E-Cigarette Aerosol with and Without THC on the Metabolic Activity of C. albicans

*C. albicans* was sub-cultured overnight at 30 °C in a 5% CO_2_ incubator. After counting with a haemocytometer, the cell suspension was adjusted to 10^6^ CFU/mL. This cell suspension was distributed in 50-mL tubes at 10^3^ CFU/mL (with a total volume of 5 mL per tube). Table 1 lists the tested conditions (which were run in duplicate). After the second e-cigarette aerosol exposure, the cell suspensions were incubated for 24 h at 30 °C in a 5% CO_2_ atmosphere. Then, the cell suspensions were centrifuged, the supernatants were discarded, and each pellet was suspended in 1 mL of fresh Sabouraud medium containing 100 µL of 3-(4,5-dimethylthiazol-2-yl)-2,5-diphenyltetrazolium bromide (MTT) solution (5 mg/mL) [47] for 3 h at 30 °C (protected from light). After this incubation, the cell suspensions were centrifuged, and the supernatants were discarded. The pellets were suspended in 1 mL of a 0.04 N HCl-isopropanol solution to release the formazan from the cell mitochondria. The cells were incubated for 15 min in the dark, under agitation, and then centrifuged for 5 min at 1200 rpm. The supernatant of each group was distributed in the wells of 96-well plates in quadruplets (four replicates with 200 µL per well), and the absorbance was read at 570 nm using an xMark microplate spectrophotometer (BioRad Laboratories, Mississauga, ON, Canada). The results are presented as the mean ± SD of five independent experiments.

### 2.7. Effect of E-Cigarette Aerosol on C. albicans Gene Expression

*C. albicans* was cultured overnight to reach the exponential growth phase. The cells were counted the cell suspension was adjusted to 10^6^ CFU/mL. This cell suspension was distributed in 50-mL tubes at 10^3^ CFU/mL (with a total volume of 5 mL per tube). Table 1 lists the different treatments. The tubes were exposed twice to e-cigarette aerosols for 5, 10, or 20 min, with a 4-h incubation period between the first and second exposures. After the second exposure, the cells were incubated for 24 h at 30 °C in a 5% CO_2_ atmosphere. Then, 10^7^ CFU of each sample was used to extract total RNA. Briefly, the cell pellet was suspended in 350 µL of lysis buffer (guanidine thiocyanate) supplemented with 14 µL of 2-mercaptoethanol and transferred to a tube containing 300 µL of sterile, RNase-free glass beads (425–600 µm size, Sigma-Aldrich Corp, St. Louis, MO, USA). The tubes were incubated for 60 min at −80 °C before bead-beating the microtubes (8–10 times, 30 s each) using a Bead Bug 6 (Benchmark, Sayreville, NJ, USA). Following cell lysis, each cell pellet was suspended in 350 µL of 70% ethanol, and total RNA was extracted using the E.Z.N.A Total RNA Kit I (Omega, BIO-TEK, Norcross, GA, USA), following the recommended protocol. Briefly, each tube was centrifuged at 12,000 rpm for 30 s. Afterward, the supernatant was discarded, and the pellets were resuspended in RNA wash buffer and centrifuged again. This process was repeated three times to ensure thorough washing. Following the final centrifugation and removal of the supernatant, the column was centrifuged to dry it and eliminate any residual ethanol. Subsequently, 40 µL of nuclease-free water was added to the column, followed by centrifugation to elute the extracted RNA. The extraction process was performed on ice at 4 °C, and the obtained RNA was stored at −80 °C. The concentration, purity, and quality of the extracted RNA were determined using NanoDrop One (Thermo Scientific, Mississauga, ON, Canada). Only those samples meeting a minimum RNA purity threshold (A260/A280) of 2.0 were accepted and used for reverse transcription quantitative polymerase chain reaction (RT-qPCR).

#### RT-qPCR

RNA (2 µg of each sample) was reverse transcribed into complementary DNA (cDNA) by using iScript Reverse Transcription SuperMix for RT-qPCR (Batch 64628077, Bio-Rad) [48]. The reaction was carried out in a MyCycler thermal cycler (Bio-Rad) with following steps: priming at 25 °C for 5 min, RT at 46 °C for 20 min, and reverse transcriptase inactivation at 95 °C for 1 min. The generated cDNA was used to measure the expression of *SAP2*, *SAP4*, *SAP9*, *EAP1*, and *HWP1* using the primers listed in Table 2. The gene sequences were cross-referenced with the *C. albicans* database and the BLAST database (Primer-BLAST, https://www.ncbi.nlm.nih.gov/tools/primer-blast/) accessed on 1 November 2024, version 2.5.0 to ensure their specificity [49]. Reactions were carried out in 96-well plates, using PCR Supermix (iQ SYBR Green Supermix, Bio-Rad) and a Bio-Rad CFX96 real-time PCR detection system. Each reaction contained 5 µL of cDNA and 15 µL of a PCR mixture consisting of 10 µL iQ SYBR Green Supermix, 0.5 µL of each primer (at a final concentration of 250 nM), and 4.5 µL RNase/DNase-free water. The thermocycling conditions were initial denaturation for 10 min at 95 °C; 40 cycles of 10 s at 95 °C (denaturation), 30 s at 60 °C (annealing), and 30 s at 72 °C (extension); 10 s at 95 °C; and a melt curve analysis run from 60 to 95 °C at a 0.5 °C increment. The specificity of each primer pair was determined by the presence of a single melting temperature peak. *S-calb*, a *C. albicans* non-encoding gene [50], showed uniform expression levels among the sample conditions (with a difference of <0.5 CT) and thus became the reference gene (housekeeping gene) for this study. The results were analysed using the 2^−ΔΔCt^ relative expression method [51]. The results are presented as the mean ± SD of three biological replicates (n = 3).

### 2.8. Statistical Analyses

Experimental values are presented as means ± SD. The statistical significance of differences between the control and the aerosol exposed test was evaluated using a one-way ANOVA. *Posteriori* comparisons were done using Tukey’s method. Normality and variance assumptions were verified using the Shapiro–Wilk test and the Brown and Forsythe test, respectively. All the assumptions were fulfilled. Data were analyzed using the SAS version 8.2 statistical package (SAS Institute Inc., Cary, NC, USA). Results were considered significant at <0.05.

## 3. Results

### 3.1. Exposure to E-Cigarette Aerosol Increased C. albicans Growth

**Figure 1:**
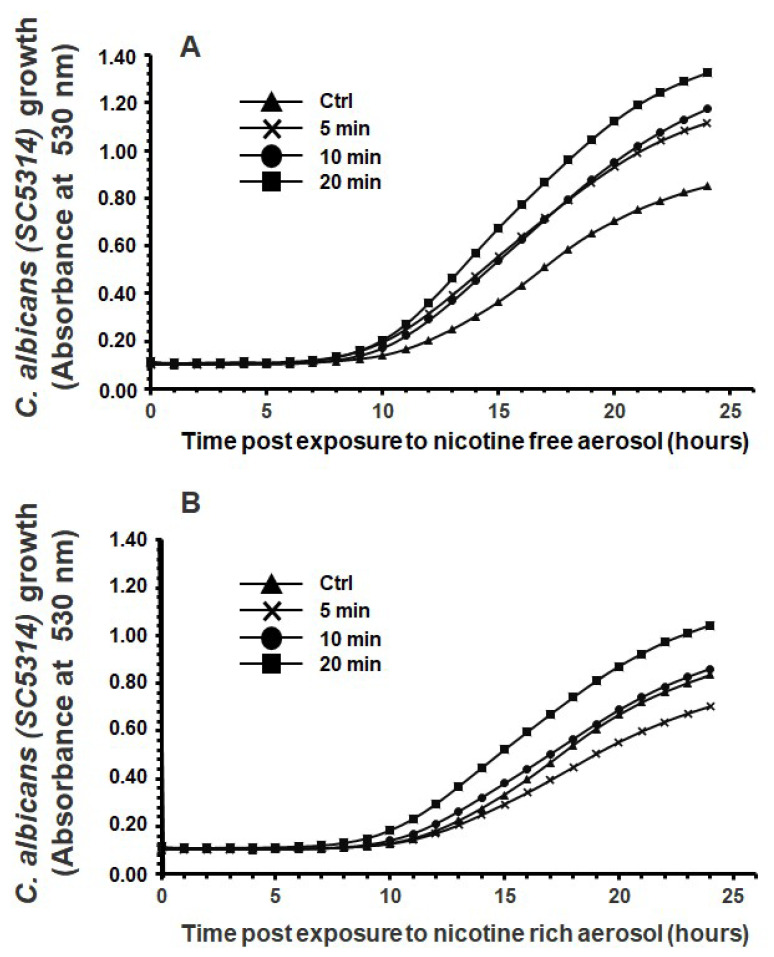
Exposure to e-cigarette aerosol (**A**) without or (**B**) with 12 mg/mL nicotine promoted the growth of *Candida albicans*. The cells were exposed twice a day to e-cigarette aerosol, and growth was evaluated by measuring turbidity at 530 nm using a spectrophotometer (n = 5).

**Figure 2:**
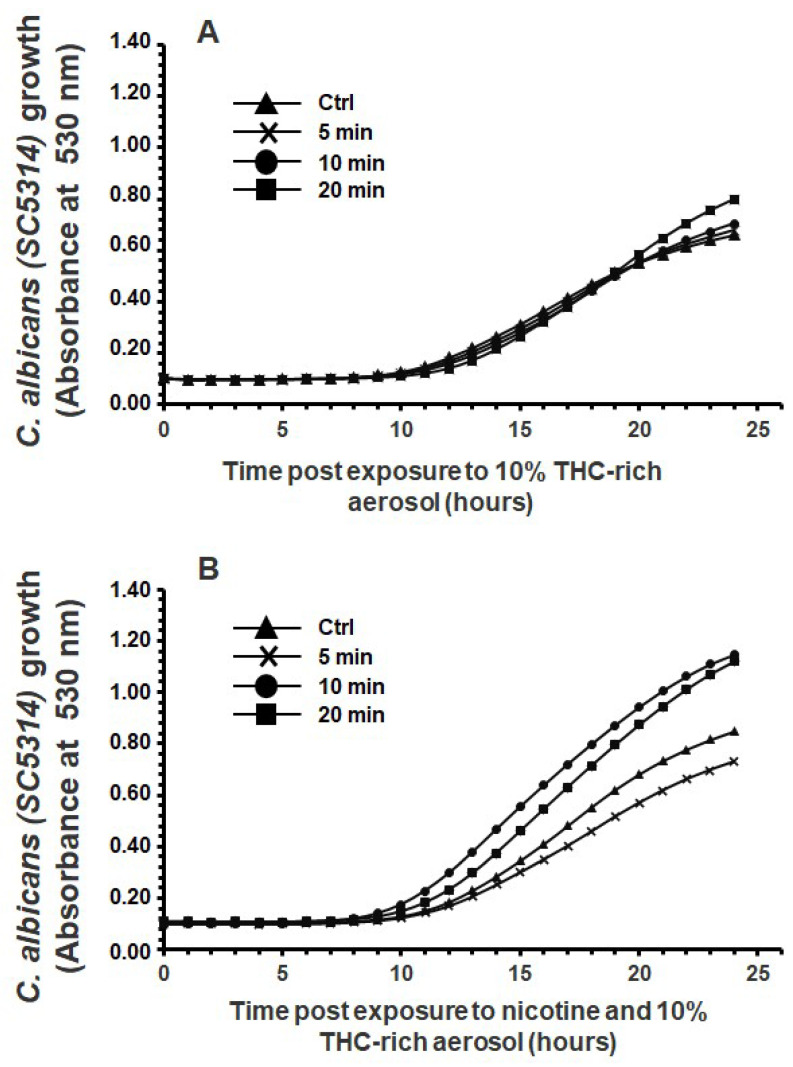
Exposure to e-cigarette aerosol with 10% tetrahydrocannabinol (THC) and (**A**) without or (**B**) with 12 mg/mL nicotine promoted the growth of *Candida albicans*. The cells were exposed twice a day to e-cigarette aerosol, and growth was evaluated by measuring turbidity at 530 nm using a spectrophotometer (n = 5).

**Figure 3:**
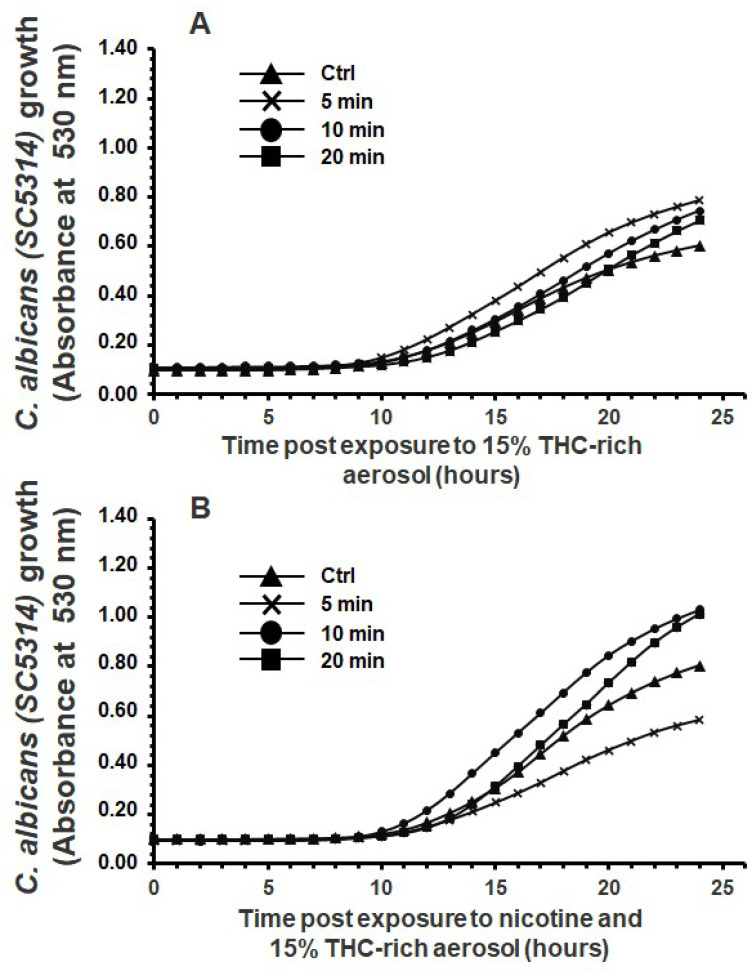
Exposure to e-cigarette aerosol with 15% tetrahydrocannabinol (THC) and (**A**) without or (**B**) with 12 mg/mL nicotine promoted the growth of *Candida albicans*. The cells were exposed twice a day to e-cigarette aerosol, and growth was evaluated by measuring turbidity at 530 nm using a spectrophotometer (n = 5).

Exposure to nicotine-free aerosol for 5, 10, or 20 min twice a day for 1 day significantly increased *C. albicans* growth starting from 15 h of contact (Figure 1A). Exposure to e-cigarette aerosol with nicotine also increased *C. albicans* growth, especially after 20 min (Figure 1B). Hence, e-cigarette aerosol with or without nicotine could increase *C. albicans* growth. Exposure to e-cigarette aerosol with 10% THC but without nicotine for 20 min twice a day slightly increased *C. albicans* growth (Figure 2A). The addition of nicotine to that aerosol significantly increased *C. albicans* growth (Figure 2B). Finally, exposure to e-cigarette aerosol with 15% THC but without nicotine for 5, 10, or 20 min twice a day significantly increased *C. albicans* growth (Figure 3A). Exposure to this aerosol containing nicotine for 10 or 20 min significantly increased *C. albicans* (Figure 3B). Taken together, exposure to vaped THC increases *C. albicans* growth.

### 3.2. Exposure to E-Cigarette Aerosol with or Without THC Increased Biofilm Formation by C. albicans

**Figure 4:**
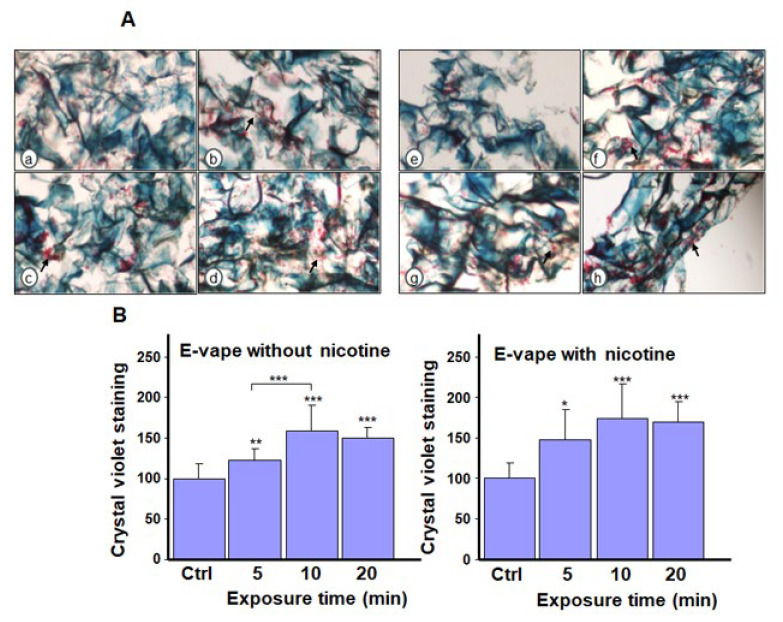
Biofilm formation by *Candida albicans* after exposure to e-cigarette aerosol with or without nicotine. The cells were exposed to e-cigarette aerosol twice a day for 3 days and then subjected to (**A**) Masson’s trichrome and (**B**) crystal violet staining. For each panel, (**a**,**e**) show the control (no exposure), (**b**,**f**) show exposure for 5 min, (**c**,**g**) show exposure for 10 min, and (**d**,**h**) show exposure for 20 min. Note that (**b**–**d**) refer to e-cigarette aerosol without nicotine, and (**f**–**h**) refer to e-cigarette aerosol with nicotine. The arrows showed *C. albicans* cell accumulation in the biofilms. * *p* < 0.05 and *** *p* < 0.001 when comparing exposure to e-cigarette aerosol to no exposure (n = 4).

**Figure 5:**
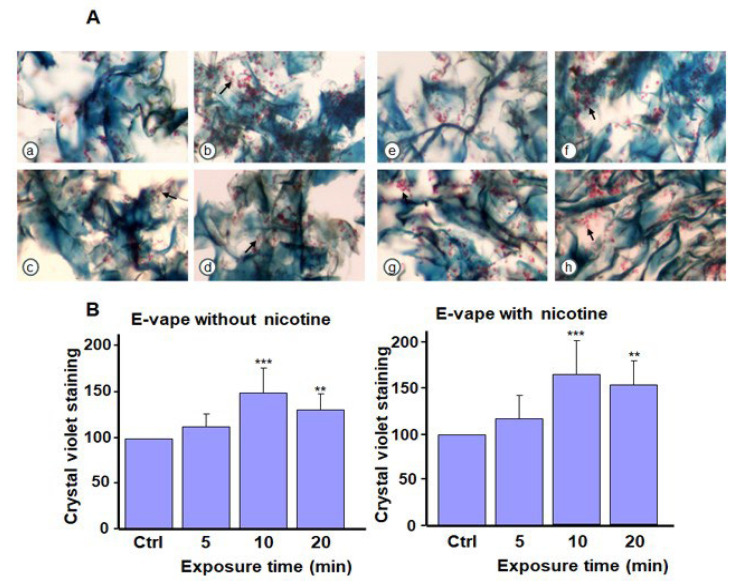
Biofilm formation by *Candida albicans* after exposure to e-cigarette aerosol with 10% tetrahydrocannabinol (THC) and with or without nicotine. The cells were exposed to e-cigarette aerosol twice a day for 3 days and then subjected to (**A**) Masson’s trichrome and (**B**) crystal violet staining. For each panel, (**a**,**e**) show the control (no exposure), (**b**,**f**) show exposure for 5 min, (**c**,**g**) show exposure for 10 min, and (**d**,**h**) show exposure for 20 min. Note that (**b**–**d**) refer to e-cigarette aerosol with 10% THC but without nicotine, and (**f**–**h**) refer to e-cigarette aerosol with 10% THC and nicotine. The arrows showed *C. albicans* cell accumulation in the biofilms. ** *p* < 0.01 and *** *p* < 0.001 when comparing exposure to e-cigarette aerosol to no exposure (n = 4).

**Figure 6:**
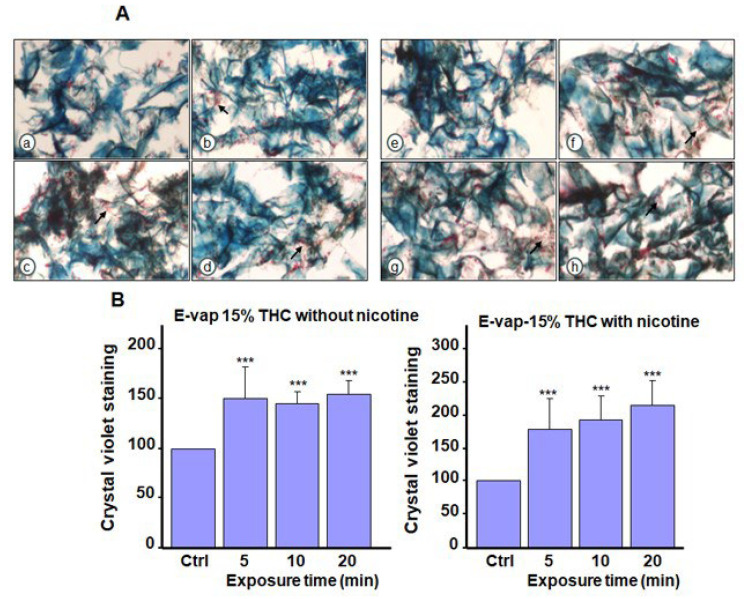
Biofilm formation by *Candida albicans* after exposure to e-cigarette aerosol with 15% tetrahydrocannabinol (THC) and with or without nicotine. The cells were exposed to e-cigarette aerosol twice a day for 3 days and then subjected to (**A**) Masson’s trichrome and (**B**) crystal violet staining. For each panel, (**a**,**e**) show the control (no exposure), (**b**,**f**) show exposure for 5 min, (**c**,**g**) show exposure for 10 min, and (**d**,**h**) show exposure for 20 min. Note that **(b**–**d**) refer to e-cigarette aerosol with 15% THC but without nicotine, and (**f**–**h**) refer to e-cigarette aerosol with 15% THC and nicotine. The arrows showed *C. albicans* cell accumulation in the biofilms. *** *p* < 0.001 when comparing exposure to e-cigarette aerosol to no exposure (n = 4).

Our histological analysis revealed a higher cell density (arrows in Figure 4A) of *C. albicans* after exposure to the e-cigarette aerosol without nicotine and THC compared with the control. The addition of nicotine led to *C. albicans* aggregation. Figure 4B presents crystal violet staining to support the histological analysis, which is indicative of biofilm formation. Exposure to e-cigarette aerosol without nicotine for 10 or 20 min twice or e-cigarette aerosol with nicotine for 5, 10, or 20 min twice a day increased biofilm formation. Histology revealed that exposure to the e-cigarette aerosol with 10% THC, with or without nicotine, twice a day increased *C. albicans* aggregations (arrows in Figure 5A). Based on crystal violet staining, exposure to e-cigarette aerosol with 10% THC and with or without nicotine for 10 or 20 min twice a day significantly increased biofilm formation (Figure 5B). Exposure to e-cigarette aerosol with 15% THC and with or without nicotine twice a day also increased *C. albicans* aggregation (Figure 6A). The crystal violet staining showed that, the exposure to e-cigarette aerosol with 15% THC and nicotine twice a day led to higher biofilm formation compared with exposure to e-cigarette aerosol with just 15% THC (Figure 6B). Taken together, exposure to e-cigarette aerosol with or without nicotine and/or THC contributes to *C. albicans* biofilm formation. Moreover, a higher THC concentration leads to more pronounced biofilm formation.

### 3.3. Exposure to E-Cigarette Aerosol with or Without Nicotine and/or THC Increased the Metabolic Activity of C. albicans

**Figure 7:**
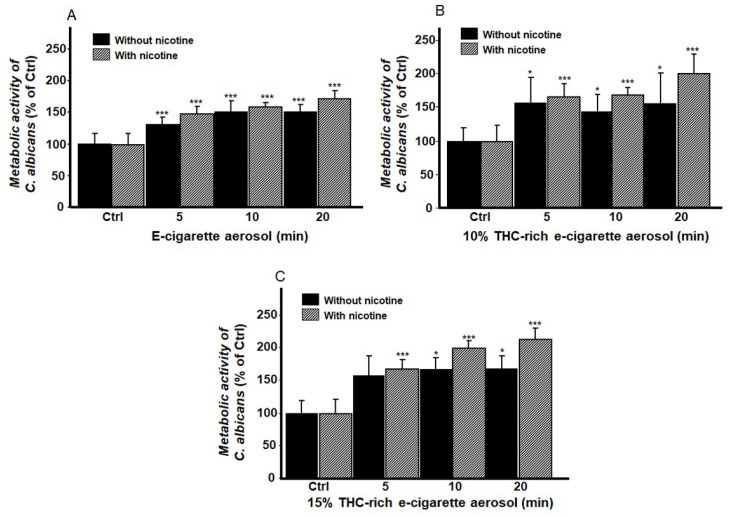
Exposure to e-cigarette aerosol increased the metabolic activity of *Candida albicans* based on the 3-(4,5-dimethylthiazol-2-yl)-2,5-diphenyltetrazolium bromide (MTT) assay. The cells were exposed to e-cigarette aerosol (**A**) with or without nicotine, (**B**) with 10% tetrahydrocannabinol (THC) and with or without nicotine, or (**C**) with 15% THC and with or without nicotine twice a day. * *p* < 0.05, *** *p* < 0.001 when comparing exposure to e-cigarette aerosol to no exposure (n = 5).

Biofilm formation by *C. albicans* can be accompanied by increased metabolic activity. Therefore, we used the MTT assay to examine metabolic activity. Exposure to e-cigarette aerosol without nicotine for 5, 10, or 20 min twice a day for one day significantly increased *C. albicans* metabolic activity (Figure 7A). The addition of nicotine to the aerosol led to greater metabolic activity (Figure 7A). Exposure to e-cigarette aerosol with 10% THC and with or without nicotine for 5, 10, or 20 min twice a day also increased *C. albicans* metabolic activity (Figure 7B). The effect was more pronounced when both nicotine and THC were present. Increasing the THC content to 15% also increased *C. albicans* metabolic activity, especially in the presence of nicotine (Figure 7C). These results demonstrate that exposure to e-cigarette aerosol with and without nicotine and with and without THC increases the metabolic activity of *C. albicans*. The higher the THC concentration, the higher the *C. albicans* metabolic activity.

### 3.4. Exposure to E-Cigarette Aerosol with or Without Nicotine and/or THC Increased the Expression of Virulence Genes

Exposure to e-cigarette aerosol without nicotine significantly induced the expression of *SAP2*, *SAP4*, *SAP9*, and *EAP1* (Table 3). Exposure to e-cigarette aerosol with nicotine for 5, 10, or 20 min twice a day also increased gene expression compared with the control. The addition of 10% THC to the e-cigarette aerosol, with or without nicotine, significantly increased the expression of each gene. The use of 15% THC without nicotine promoted the expression of the virulence genes (Table 3). Exposure to the e-cigarette aerosol with 15% THC-rich aerosol and nicotine led to higher gene expression compared with exposure to the e-cigarette aerosol with 15% THC but without nicotine. Overall, these results demonstrate that exposure to e-cigarette aerosol promote the expression of genes involved in *C. albicans* growth and biofilm formation.

## 4. Discussion

Vaping cannabinoids such as THC could have adverse effects on the entire body, including the oral cavity. The entry of cannabis-rich e-cigarette aerosol into the mouth could perturb the microbial ecosystem [52], leading to oral infection, including candidiasis. In this study, we investigated the effect of e-cigarette aerosol with or without nicotine and/or THC on the growth of *C. albicans*. We first demonstrated that exposure to e-cigarette aerosol with or without nicotine promoted the growth of *C. albicans*, especially after exposure for 10 or 20 min twice a day. These results are consistent with a previous report on *C. albicans* growth following a 15-min exposure [45]. It has also been reported that oral *C. albicans* carriage is significantly higher in e-cigarette users than never-smokers [53]. The addition of 10% or 15% THC to the e-cigarette aerosol significantly increased *C. albicans* growth, with a greater increase with the exposure for 10 or 20 min twice a day. This study is the first to show an effect from exposure to vaped THC on *C. albicans*, suggesting a possible link between vaping cannabis derivatives and oral infection [54]. The use of e-cigarettes containing THC has also been reported to increase the risk of dental caries [29]. The increased growth of *C. albicans* following exposure to e-cigarette aerosol with THC was accompanied by increased biofilm formation. This observation supports a previous study showing that exposure of *C. albicans* to e-cigarette condensate increases biofilm formation [55]. Note that this effect is not limited to *C. albicans*, as e-cigarettes have also been reported to increase biofilm formation of the pathogen *Streptococcus mutans* [56,57].

Based on prior research, smoking cannabis has negative effects on the gums [58] and teeth [59] as well as the oral ecosystem [60]. An increased prevalence of *C. albicans* in cannabis users has also been shown compared with non-smoking controls [60]. Furthermore, an in vitro study demonstrated the contribution of cannabis smoke to *C. albicans* biofilm formation [61]. Consistent with the results from the present study, *C. albicans* biofilm formation could be promoted by vaping cannabis derivatives, including THC. The biofilm formation by *C. albicans* may utilise the various chemicals that are generated after THC vaping [27,62].

*C. albicans* growth and biofilm formation are closely linked to metabolic activity [63]. Because we demonstrated increased *C. albicans* growth and biofilm formation following exposure to e-cigarette aerosol with THC, we evaluated metabolic activity with the MTT assay. We showed for the first time that exposure to e-cigarette aerosol with THC increases *C. albicans* metabolic activity. These results support reports of increased *C. albicans* growth and metabolic activity after exposure to cigarette smoke [64] and cannabis smoke [61].

*C. albicans* growth and biofilm formation are regulated by numerous genes [65]. Among these, we examined the expression of *SAP2*, *SAP4*, and *SAP9*, as they are involved in *C. albicans* growth and virulence [66]. The expression of these genes increased after exposure to nicotine and THC-free e-cigarette aerosols. Such an effect could be attributed to bioproduct generated from heated PG, VG, or PG/VG products, as non-vaped PG showed anti-*C. albicans* activity [67]. This observation warrants further study to validate this hypothesis.

Interestingly, exposure to aerosol with THC but without nicotine increased the expression of *SAP2*, *SAP4*, and *SAP9*, which was higher after exposure for 5 min compared with 20 min. It appears that the longer *C. albicans* is exposed to aerosolised THC, the lower the expression of *SAP2*, *SAP4*, and *SAP9*. Such an observation could be explained by an adaptation of *C. albicans* to this exposure condition, leading to basal expression of *SAP* genes [68]. Also, the exposure to aerosol, whatever its content, could lead to yeast stress modulating the genes `expressions, as previously reported [69]. Additional studies are needed to test this hypothesis.

Exposure of *C. albicans* to e-cigarette aerosols with and without nicotine and THC also increased *EAP1* expression. As a member of the GPI-CWP family, EAP1 promotes the adhesion of *C. albicans* to different surfaces [70]. This suggests that exposure to e-cigarette aerosol with or without nicotine could contribute to *C. albicans* growth and biofilm formation by promoting adhesion and environmental adaptation. Interestingly, we have demonstrated for the first time that exposure to e-cigarette aerosol with THC and with or without nicotine contributed to a significant increase in the EAP1 gene expression, especially with 15% THC. Semlali et al. (2014) reported similar observations with cigarette smoke [48], but there had been no previous investigation as to whether cannabis smoking or vaping produced similar changes.

This study has some limitations that should be noted. First, although C. albicans is the most frequent yeast causing oral candidiasis, other Candida species can infect the oral cavity. Indeed, *Candida glabrata* is also a prevalent pathogen in oral diseases [71]. It has been reported that patients receiving chemotherapy for leukemia, *Candida tropicalis* causes oropharyngeal candidiasis and candidemia [72]. Furthermore, in the last two decades, other Candida species, including *C. parapsilosis* and *C. krusei*, have been identified as causing human diseases such as oropharyngeal candidiasis [73]. These non-*C. albicans* species can also be in contact with e-cigarette aerosol, contributing to oral infections. Another limitation of our study is the use of an in vitro model. Although very helpful to generate transposable data for humans, the results need to be validated with an animal model combining the exposure to e-cigarette aerosols with and without THC and *Candida* species to mimic what could happen with cannabis-rich e-cigarette users closely. Another potential limitation is the lack of use of a *C. albicans* clinical isolate in this study. Thus, future studies should include such clinical isolates to reflect on what could happen in e-cigarette users who are carrying *C. albicans*. After vaping, potential thermal decomposition of the e-liquid constituents (PG, VG, THC) could occur, leading to deregulation of yeast behaviors. Finally, when translating this study to e-cigarette users, attention should also be paid to the potential combination of THC-vaping along with cannabis and or cigarette smoke.

## 5. Conclusions

Exposure of *C. albicans* to e-cigarette aerosol containing THC affects the growth, biofilm formation, and metabolic activity of this fungus. It also leads to high expression of genes involved in *C. albicans* adhesion, growth, and biofilm formation. Our results suggest that vaping THC could promote oral candidiasis in e-cigarette users. Oral health providers should be aware and systematically question patients about their cannabis derivative vaping habits to offer the best oral care.

## Figures and Tables

**Table 2:** Primer sequences used in the qRT-PCR.

Gene	Name	Primer Sequence 5′ to 3′	Size (bp)
*s-Calb*	Noncoding gene	F: ATGTGGCACGGCTTCTGCTG R: TAGGCTGGCAGTATCGTCAGAGG	53
*SAP2*	Secreted Aspartyl Proteinase 2	F: TCCTGATGTTAATGTTGATTGTCAAG R: TGGATCATATGTCCCCTTTTGTT	82
*SAP4*	Secreted Aspartyl Proteinase 4	F: CAATTTAACTGCAACAGGTCCTCTT R: AGATATTGAGCCCACAGAAATTCC	82
*SAP9*	Secreted Aspartyl Proteinase 9	F: ATTTACTCCACAGTTTATCACTGAAGGT R: CCACAAGAACCACCCTCAGTT	86
*EAP1*	Enhanced Adhesion Protein 1	F: CTGCTCACTCAACTTCAATTGTCG R: GAACACATCCACCTTCGGGA	51

**Table 3:** Genes ’expressions following the exposure of *C. albicans* to e-cigarette aerosols.

	Exposure Time to E-Cigarette Aerosol			
	Ctrl	5 min	10 min	20 min
Exposure to nicotine-free aerosol				
SAP2	1 ± 0.00	11.9 ± 0.2 ***	1.5 ± 0.2 ***	1.7 ± 0.1 ***
SAP4	1 ± 0.00	1.6 ± 0.2 ***	1.4 ± 0.3 **	1.6 ± 0.2 ***
SAP9	1 ± 0.00	2.3 ± 0.1 ***	2.1 ± 0.1 ***	2 ± 0.1 ***
EAP1	1 ± 0.00	1.4 ± 0.1 ***	1.57 ± 0.07 ***	1.8 ± 0.05 ***
Exposure to nicotine-rich aerosol				
SAP2	1 ± 0.00	1.23 ± 0.05 ***	1.19 ± 0.1 ***	1.3 ± 0.1 ***
SAP4	1 ± 0.00	1.0 ± 0.03	1.08 ± 0.04	1.2 ± 0.09 *
SAP9	1 ± 0.00	1.4 ± 0.03 ***	1.3 ± 0.05 ***	1.6 ± 0.1 ***
EAP1	1 ± 0.00	1.5 ± 0.02 ***	1.5 ± 0.05 ***	1.47 ± 0.1 ***
Exposure to 10% THC-rich aerosol without nicotine				
SAP2	1 ± 0.00	1.6 ± 0.011 ***	1.35 ± 0.02 ***	1.08 ± 0.05 *
SAP4	1 ± 0.00	1.7 ± 0.02 ***	1.6 ± 0.08 ***	1.7 ± 0.09 ***
SAP9	1 ± 0.00	1.6 ± 0.1 ***	1.3 ± 0.09 ***	1.2 ± 0.01 ***
EAP1	1 ± 0.00	1.8 ± 0.05 ***	1.6 ± 0.06 ***	1.6 ± 0.09 ***
Exposure to 10% THC and nicotine-rich aerosol				
SAP2	1 ± 0.00	1.1 ± 0.02	1 ± 0.05	1.3 ± 0.03 ***
SAP4	1 ± 0.00	1 ± 0.01	1.4 ± 0.05 ***	2 ± 0.07 ***
SAP9	1 ± 0.00	1.7 ± 0.1 ***	1.6 ± 0.09 ***	1.3 ± 0.09 ***
EAP1	1 ± 0.00	1.4 ± 0.1 ***	1.3 ± 0.1 ***	1.2 ± 0.03 ***
Exposure to 15% THC-rich aerosol without nicotine				
SAP2	1 ± 0.00	1.08 ± 0.001	1.1 ± 0.02 *	1.15 ± 0.04 ***
SAP4	1 ± 0.00	1.3 ± 0.02 ***	1.4 ±0.07 ***	1.7 ± 0.08 ***
SAP9	1 ± 0.00	1.3 ± 0.07 ***	1.4 ± 0.06 ***	1.4 ± 0.09 ***
EAP1	1 ± 0.00	1.7 ± 0.06 ***	1.6 ± 0.05 ***	1.52 ± 0.098 **
Exposure to 15% THC and nicotine-rich aerosol				
SAP2	1 ± 0.00	1.3 ± 0.07 ***	1.2 ± 0.07 ***	0.8 ± 0.3
SAP4	1 ± 0.00	1.45 ± 0.05 ***	1.5 ± 0.08 ***	2 ± 0.09 ***
SAP9	1 ± 0.00	2.5 ± 0.1 ***	2 ± 0.09 ***	3.1 ± 0.1 ***
EAP1	1 ± 0.00	2.3 ± 0.1 ***	2.5 ± 0.06 ***	4.6 ± 0.2 ***

Statistical significance was obtained when comparing exposure to e-cigarette aerosol to no exposure (Ctrl) (n = 3). *p* value (*p*) = (*) 0.05, (**) 0.01, and (***) 0.001. Ctrl = control.

## Data Availability

All data generated or analyzed during this study are included in this published article.
